# The Value of Engaging the Public in CHATing About Healthcare Priorities: A Response to Recent Commentaries

**DOI:** 10.15171/ijhpm.2018.113

**Published:** 2018-12-02

**Authors:** Marion Danis, Susan D. Goold, Melinee Schindler, Samia A. Hurst

**Affiliations:** ^1^Department of Bioethics, National Institutes of Health, Bethesda, MD, USA.; ^2^Department of General Internal Medicine, University of Michigan Medical Center, Ann Arbor, MI, USA.; ^3^Institute for Ethics, History, and the Humanities, Geneva University Medical School, Geneva, Switzerland.

Leonard Fleck and Ann Mongoven have each commented on our publication of a project in which we engaged members of the Swiss public in a priority-setting exercise deliberating about what should be included in (mandatory) health insurance packages.^1,2^ The project made use of the Choosing Healthplans All Together (CHAT) exercise – an exercise that allows small groups to deliberate about and choose coverage over approximately 3 hours. Both Fleck and Mongoven acknowledge that there are strengths of the exercise – as Fleck comments, it is engaging, thought-provoking and well designed. But we will focus here on replying to the concerns and questions that they each raised.

Fleck articulates three concerns. First, he is concerned that priority setting is complex and cannot be dealt with thoroughly in a brief engagement. When MD and SG were first designing the CHAT exercise, this was done specifically to confront the complexity – and the salience – of policy options. The exercise intentionally presents complicated decision-making tasks in an understandable and engaging manner so that the public *can* work in groups to effectively make choices together. We would emphasize that while the presentation of options in the exercise is simple, the preparatory work that underlies the options that are presented in a CHAT exercise involves very detailed background work. We work with various informants (including policy-makers, healthcare experts and, at times, community partners) to decide which options to include in the exercise, how to describe them in an accessible and credible way, and how to best illustrate the tradeoffs with “events.”^3^ Actuarial analyses which involve estimation of the probability that the interventions are likely to be used and calculation of the probable expense of covering these interventions, are carried out. In this way the CHAT exercise combines involvement and ascertainment of the views of non-professional members of the public with evidence-based expertise. Second, we would not propose that the exercise should be the only source of public input on complex issues that will drive policy decisions. Other deliberative techniques, for instance those that involve several days of intense exposure to information and deliberation by a small number of selected individuals, might also, or more appropriately be used depending on the decision(s) to be made. CHAT projects often enroll hundreds of participants in dozens of small deliberative groups (often geographically dispersed), while longer, more intense deliberations may be limited by location or the availability of people willing to serve long-term. While we would not suggest that CHAT is necessarily sufficient on its own to incorporate the public voice in policy decisions, we do believe it adds a very important dimension that ought not to be missed.

Second, Fleck is concerned about the ‘liberalism problem’ – the concern that diverse views will be drowned out by the majority in the course of the deliberation. We would suggest, first, that this could happen (and does happen) in many types of public deliberation, and, second, the CHAT exercise can help mitigate that risk. The CHAT exercise, like some other deliberative processes, involves a trained facilitator with explicit instructions about the need for reason-giving and the need to hear and respect all participant points of view. CHAT also allows for the ascertainment of individual choices along with group choices. How the choices that reflect minority opinions are accommodated when policies are actually made is a different question that is not addressed by the CHAT exercise. It remains for policy-makers to decide what to do with the results of a public deliberation. For example, when designing health insurance coverage packages, policy-makers can offer a larger number or a smaller number of policy coverage options which are thus more or less respectful of diverse preferences among the population.

Third, Fleck states that ‘our sense of justice’ is complex and context dependent. He describes several complicated situations that require attention to particular details to achieve a thorough analysis and sound conclusions. We might address this concern in either of two ways. First, to the extent that the CHAT exercise can be tailored to address a more focused priority setting problem, one could narrow the focus of a CHAT exercise and hone in on particular details in greater depth. Alternatively, we would suggest that the narrower the focus of priority setting, the more risk there is that tradeoffs will not be viewed as necessary or credible. If people are asked, for instance, to set priorities for the treatment of leukemia, they may well complain that resources from other needs could make that unnecessary (or easier). CHAT addresses priorities and tradeoffs with a broader lens. Rather than focusing on whether to insure treatment for a singular particular rare disease, we might frame the choice in general terms: should more funds be spent on widely prevalent diseases or on the treatment of rare conditions. With this more general framing, and with examples used to illustrate the consequences of those tradeoffs, the ethical tension between prioritizing the needs of many versus the needs of the few can be clearly appreciated and debated by participants in the exercise. It is true that broad principles agreed to in theory may not be accepted in practice when facing an individual patient’s need. This, however, applies to the more general conclusions of priority setting when applied to individual cases, with or without input from public deliberation. This general difficulty does not make the identification of such broad principles, or public participation in the process, any less important and interesting.

Both Fleck and Mongoven express concerns about how the results of CHAT exercises ought to be translated into policy. It has not been our intention that the results of CHAT exercises be translated into priority setting decisions directly, any more than those who argue for the consideration of cost-effectiveness would argue that those results alone determine coverage priorities. Nor would such an automatic process be likely since policy-making always involves many negotiations and considerations on the part of those in charge of budgeting.

Mongoven points out that preparation of the CHAT board might itself require a public deliberative process. We agree. In fact, several CHAT projects have included a participatory process with community leaders and other stakeholders.^4,5^

Mongoven asks how a facilitator of a CHAT exercise can address the challenge of supporting without directing deliberation. A facilitator guides the group discussion by inviting participants to each take a turn voicing a coverage choice and explaining the choice. After each choice is expressed, the group has a chance to comment on the choice, accept it or change it. If the group comes to consensus, the markers are placed on the CHAT board accordingly. If there is no consensus, the group may return to that option later, or decide to vote on how to allocate funds to the choice(s) proposed. Occasionally a participant will mention a point that is factually incorrect. A facilitator might correct the factual error but is not supposed to guide the discussion otherwise (a facilitator script is available from the authors on request).

Mongoven asks whether a theory-light game could travel light to diverse political and organizational contexts. Could it be employed at pressure points for micro-distributional justice as well as macro (for example, within a specific insurance pool)? Could it be adapted to promote civic discussion in schools among countries that have different health care financing and delivery systems? We would point out that the CHAT exercise is designed to be modifiable so it can be used in such diverse contexts: the priority setting task, the coverage options, the population to be covered, the language in which the exercise in conducted, and the exercise materials can be changed,^6^ and that it has been used in a wide variety of contexts and settings.^6,7^

While the comments above largely related to the CHAT process, Mongoven also comments about the substantive results of the Swiss CHAT exercise. As she notes, the participants in the exercise tended to endorse current health insurance coverage policy in Switzerland and asks why this might be. It is important to note that they did not support the status quo in all areas of health coverage. While the changes proposed did not affect a majority of the areas within the game, some would, if implemented, lead to significant changes in the delivery of healthcare in Switzerland. As Mongoven notes, the Swiss-CHAT exercise also enabled an inversion of the majority view on whether the primary goal of insurance is to cover routine health costs (initial majority view) or to address unpredictable serious illnesses and crises (post-exercise majority view).

Mongoven also wonders to what extent individuals found the group choice acceptable. We generally ask respondents at the conclusion of CHAT exercises, “Would you be willing to abide by the decision made by your group?” and usually find at least 85% say yes.^6^ More in-depth analysis of our data also shows our participants to have reasoned in a strategic manner to attempt prudent priority setting for the health care system as a whole. They took the plight and potential plight of others into account, and made decisions based on solidarity and cost-containment.^8^ Such reasoning could have made them more likely to find the group decision acceptable at the end of the exercise.

Mongoven also notes that the exercise brought out some particular findings that are encouraging: Many participants were willing to accept the need to make trade-offs and set priorities, and many viewed health insurance coverage offered in Switzerland sufficient. They would not opt for additional coverage.

We do appreciate that both Fleck and Mongoven hope that experiences like CHAT can encourage, catalyze or improve capacity for other public deliberation. To quote Mongoven, “theory may be less important than getting people in the game.” We, too, find the ease with which citizens discussed these complex issues, and the encouragement it affords for public participation in health care decisions, to be one of the more important findings here.

## Acknowledgements

This work was supported in part by the intramural program at the National Institutes of Health, by the Brocher Foundation, and by the Swiss Academy of Medical Sciences.

## Ethical issues

Not applicable.

## Competing interests

Dr. Goold, Dr. Danis, and their institutions may benefit from royalties paid by future users of CHAT. The authors declare that they have no other competing interests. The views expressed here are those of the authors and do not necessarily reflect the policies of the institutions at which they work.

## Authors’ contributions

MD, SDG, MS, and SAH contributed to the conception and design of the Swiss-CHAT study, to the analysis and interpretation of data, and to revisions of this manuscript for important intelectual content. MD wrote the first draft. All authors have approved the final version.

## Authors’ affiliations

^1^Department of Bioethics, National Institutes of Health, Bethesda, MD, USA. ^2^Department of General Internal Medicine, University of Michigan Medical Center, Ann Arbor, MI, USA. ^3^Institute for Ethics, History, and the Humanities, Geneva University Medical School, Geneva, Switzerland.
